# Analysis of Microbial Community Structure and Cultivation Performance Assessment in *Tremella fuciformis* Liquid Inoculum

**DOI:** 10.3390/jof11120825

**Published:** 2025-11-22

**Authors:** Hui Lin, Qi Xiong, Wenxuan Huang, Xinghua Dai, Yingxi Yang, Wenlin Huang, Shufang Lai, Shujing Sun, Liaoyuan Zhang

**Affiliations:** 1 Institute of Edible Mushroom, Fujian Academy of Agricultural Sciences, Fuzhou 350002, China; linhui-syjs@faas.cn (H.L.); 52405043097@fafu.edu.cn (Q.X.); 52305043034@fafu.edu.cn (W.H.); 12305014021@fafu.edu.cn (X.D.); 52305043035@fafu.edu.cn (Y.Y.); 12405014018@fafu.edu.cn (W.H.); 2 College of Life Sciences, Fujian Agriculture and Forestry University, Fuzhou 350002, China; 3 Fujian Edible Fungus Technology Promotion General Station, Fuzhou 350002, China; 52305044025@fafu.edu.cn

**Keywords:** *Annulohypoxylon stygium*, liquefied spawn, liquid spawn, practical application evaluation, quality dynamics, fruiting body yield

## Abstract

Liquid inoculum is widely adopted in the mushroom industry, yet preparing *Tremella fuciformis* liquid inoculum remains challenging due to its complex microbial community and dimorphic growth. This study aimed to establish a reliable protocol for *T. fuciformis* liquid inoculum and assess its practical application. Initially, liquefied spawn was produced by liquefying solid spawn. The application of standard liquefied spawn increased fruiting body yield by 8.2% (502.4 g/kg dry substrate) compared to solid spawn, but exhibited substantial batch-to-batch variation due to unstable microbial communities and low *Tremellomycetes* abundance. To address these limitations, liquid spawn was developed via pre-culture of pure *T. fuciformis* and *Annulohypoxylon stygium* mycelia. Cultivation tests demonstrated significantly enhanced performance with 608.2 g of fruiting bodies, which represented 11.1% improvement compared to solid spawn. Moreover, consistent yields could be observed across multiple batches. This stability was attributed to stable microbial community structure and the dominance of *Tremellomycetes* (abundance > 50%) in the fungal community. These results confirm the cultivation performance of *T. fuciformis* liquid spawn, highlighting its potential as an effective alternative to solid spawn for *T. fuciformis* industrial production.

## 1. Introduction

*Tremella fuciformis* is commonly known as white jelly mushroom, snow ear, or silver ear fungus, and belongs to the class *Tremellomycetes*, order *Tremellales*, and family *Tremellaceae*. As a typical dimorphic fungus, it exhibits two distinct morphological transitions: (i) the yeast-to-pseudohyphae transition and (ii) the yeast-to-hyphae transition [1,2]. The fruiting body of *T. fuciformis* contains abundant nutritional components, notably polysaccharides, proteins, dietary fibers, vitamins, and essential mineral elements [3]. Otherwise, *T. fuciformis* has demonstrated significant utility in both cosmeceutical and pharmaceutical applications owing to its diverse biological activities, including antioxidant, immunomodulatory, neuroprotective, and antitumor activities [4,5,6]. *T. fuciformis* is a geographically widespread fungus, frequently colonizing decaying broadleaf tree branches in tropical regions [7]. Although initially discovered in Brazil, industrial scale cultivation has established it as a major commercially cultivated species in China, with total production value reaching approximately 25.5 billion RMB (about 3.5 billion USD) in 2024 [8]. The industrial cultivation process employs a standardized substrate formulation comprising primarily wheat bran and cottonseed hulls, with *T. fuciformis* harvested on a bimonthly basis. This industrialized method dominates *T. fuciformis* production, accounting for over 90% of the global market.

In all environments including cultivation conditions, the fruiting body formation of *T. fuciformis* is obligately dependent on its companion ascomycete *Annulohypoxylon stygium* [9]. *A. stygium* is a white-rot filamentous fungus belonging to the family Xylariaceae [10], which can be routinely isolated from both woody substrates adjacent to *T. fuciformis* basidiomes and spawn cultures used in cultivation systems. As an associated fungus, *A. stygium* exhibits exceptional enzymatic capability in degrading lignin and carbohydrates, thereby generating essential nutrients that support *T. fuciformis* fruiting body development [11,12]. During the artificial cultivation of mushrooms, inoculation of the spawn into the substrate constitutes a crucial procedural step. Currently, solid spawn remains the standard inoculum in *T. fuciformis* cultivation protocols. Prior to inoculation, the solid spawn must undergo thorough homogenization to guarantee that each inoculation unit contains viable hyphae of both *T. fuciformis* and *A. stygium*. However, this method faces critical limitations, including high technical difficulty in spawn preparation, prolonged spawn running time [13], high contamination risk, poor stability of solid spawn preparation [14], and pronounced spawn degeneration [15]. These factors collectively contribute to batch-to-batch variability in the yield and quality of *T. fuciformis*, posing a major bottleneck for standardized, large-scale production.

Liquid inoculum has been developed for more than 30 years, with liquefied spawn and liquid spawn representing the two most prevalent forms. The former is produced by liquification of solid mother spawn, whereas the latter is obtained through the pure liquid culture of mycelium. These inoculum types are garnering heightened attention from mycologists and commercial cultivators due to its multifaceted advantages including shortened preparation period, reduced space requirements, lower production costs, rapid substrate colonization capacity, compatibility with automated inoculation equipment, and facilitated uniform dispersion within growth substrates [16,17]. Numerous commercially important edible mushrooms such as *Lentinula edodes* [18], *Pleurotus eryngii* [19], *Ganoderma lucidum* [20] and *Flammulina filiformis* [21], have successfully developed and implemented standardized industrial-scale protocols for liquid inoculum production. High-quality liquid inoculum enhances edible mushroom cultivation efficiency by reducing the production cycle and simultaneously boosting yield and fruiting body quality [22]. However, *T. fuciformis* lacks both standardized liquid spawn protocols and systematic studies on this cultivation method. This gap is particularly challenging due to the unique dimorphic growth characteristics of *T. fuciformis* and the markedly divergent growth dynamics with its companion *A. stygium* in liquid culture systems, which introduce significant technical barriers to liquid culture development [23,24]. Addressing this critical knowledge gap in mycological cultivation research is imperative to establish fundamental biological understanding and develop optimized liquid culture protocols for this species.

This study systematically investigated two distinct preparation methods for *T. fuciformis* liquid spawn: (1) solid spawn liquefaction and (2) pure mycelial liquid culture. Cultivation experiments were conducted to validate their application feasibility. Microbial community structures in both types of liquid spawn were comparatively analyzed through high throughput sequencing, analyzing the fluctuations in microbial community structures and fruiting body yield across multiple liquid spawn batches to estimate the application performance. The findings provide both theoretical foundations and practical references for standardizing, orienting, and scaling up industrial production of *T. fuciformis* liquid spawn, while proposing innovative strategies to enhance the sustainable development of the entire *T. fuciformis* cultivation industry.

## 2. Materials and Methods

### 2.1. Chemicals and Reagents

Analytical Reagent including glucose (CAS Number: 50-99-7), KH_2_PO_4_ (CAS Number: 7778-77-0), MgSO_4_ (CAS Number: 7487-88-9), K_2_HPO_4_ (CAS Number: 7758-11-4), absolute ethanol (CAS Number: 64-17-5), phenol (CAS Number: 108-95-2), sulfuric acid (CAS Number: 7664-93-9), NaOH (CAS Number: 1310-73-2), HCl with a concentration of 36.0–38.0% (CAS Number: 7647-01-0) and agar powder (CAS Number: 9002-18-0) were purchased from Sinopharma Chemical Reagent Co., Ltd. (Shanghai, China). Thiamine HCl (CAS Number: 67-03-8) was bought from Sigma Aldrich (Shanghai, China). Oxoid™ Yeast Extract Powder (Code: LP0021) and Tryptone (Code: LP0042) were purchased from Thermo Fisher Scientific (Waltham, MA, USA). Potato, corn flour, and soybean flour were obtained from a local supermarket (Fuzhou, China). The cultivation substrate components including cottonseed hull, wheat bran, and gypsum were purchased from a specialized market for edible mushroom raw and auxiliary materials in Gutian, Ningde (Fujian, China).

### 2.2. Strains and Culture Conditions

The *Tremella fuciformis* strain Tr21 and its companion fungus *Annulohypoxylon stygium* were isolated from high-quality fruiting bodies of *T. fuciformis* (Jianhong Agricultural Development Co., Ltd., Ningde, China), and subsequently identified through ITS region amplification and sequencing analysis. Both strains were separately cultured on PDA plates (potato 200 g/L, glucose 20 g/L, peptone 5 g/L, KH_2_PO_4_ 2 g/L, MgSO_4_ 1 g/L, agar 20 g/L, Thiamine HCl 0.01 g/L) at 25 °C. The cultures were cryopreserved at −80 °C with the addition of 30% glycerol at the Gutian Edible Fungi Research Institute, Fujian Agriculture and Forestry University, Fuzhou, China.

### 2.3. The Method of T. fuciformis Liquefied Spawn Preparation

The liquefied spawn of *T. fuciformis* was prepared by modifying established literature protocols [25]. Mother spawn of *T. fuciformis*, obtained from a pure co-culture of *A. stygium* and *T. fuciformis* mycelia on a lignocellulose substrate in glass bottles, characterized by round white mycelial aggregates on the substrate surface and vigorous hyphal growth of *A. stygium*, was selected for liquefied spawn preparation. A 3 cm-deep substrate layer beneath the surface was homogenized using a sterile mixer (Zhongchong (Jining, China), TJ3). Subsequently, 50 g of the homogenized substrate was mixed with 150 mL of sterile water (1:3, *w*/*v*). The mixture was ground at 10,000 rpm for 40 s under aseptic conditions using a knife mill homogenizer (Retsch (Haan, Germany), GRINDOMIX GM 300) to prepare the mother suspension. Aliquots (0.3 g) of the mother suspension were inoculated into liquefied spawn media I-VII (Table 1). These cultures were incubated at 24 °C and 140 rpm for 4 days to produce the *T. fuciformis* liquefied spawn. The optimal medium was screened based on mycelial pellet density and average pellet diameter. Additionally, the effects of inoculum amount of mother suspension (0.1–0.9 g), grinding time (30–90 s), medium volume (50–150 mL), and initial medium pH (4–9.5) on the quality of liquefied spawn were evaluated. Mycelial pellet density was determined by transferring 5 mL of liquefied spawn into a transparent Petri dish and counting the pellets under 10× magnification (five replicates). Mycelial pellet diameter was assessed by linearly aligning ten randomly selected pellets using an inoculating needle and measuring the total length to calculate the mean diameter (five replicates).

### 2.4. Physiological Parameter Assay of T. fuciformis Liquefied Spawn

The physiological parameters of *T. fuciformis* liquefied spawn during the cultivation process were measured for the optimal incubation time. Morphological indicators, including mycelial pellet growth performance and changes in broth color, were macroscopically observed. Four key parameters including pH value, biomass, soluble protein and glucose concentration were quantitatively analyzed. The detailed procedures were as follows: 20 mL of *T. fuciformis* liquefied spawn culture was transferred to a 50 mL beaker, and the pH value was measured using a pH meter (Mettler-Toledo (Changzhou, China), FE28). For biomass determination, 10 mL of the culture was centrifuged at 10,000× *g* rpm for 20 min to collect mycelial pellets from the supernatant. The harvested mycelial pellets were washed twice with deionized water. Excess moisture was removed using filter paper, and the mycelia were dried to a constant weight in an oven at 55 °C. The supernatant was used for further analysis of soluble protein and glucose concentration. Soluble protein concentration was quantified using the Bradford method, while glucose concentration was determined via the DNS (3,5-dinitrosalicylic acid) method [26].

Melanin and polysaccharide content are critical quality indicators for *T. fuciformis* strains. The detailed protocols for analyzing intracellular and extracellular polysaccharides, as well as melanin content in *T. fuciformis* liquefied spawn cultures, are outlined below: Intracellular and extracellular polysaccharides were obtained from *T. fuciformis* culture medium according to the method of Liu et al. [27]. For extracellular polysaccharide analysis, 10 mL of culture was subjected to high-speed centrifugation (10,000× *g* rpm, 10 min) to collect the supernatant. The supernatant was concentrated to 2 mL using a rotary evaporator (Shanghai Yarong (Shanghai, China), RE-52AA), followed by precipitation with three volumes of absolute ethanol. The mixture was vortexed thoroughly and stored at 4 °C for 10–12 h to allow complete polysaccharide precipitation. Precipitates were collected by centrifugation (10,000× *g* rpm, 10 min), dissolved in distilled water, and re-centrifuged to remove insoluble residues. For intracellular polysaccharide analysis, mycelia were harvested from 10 mL of culture by centrifugation and washed twice with sterile water. Cell lysis was achieved by adding six volumes (*v*/*w*) of sterile water to the mycelial pellet, followed by three freeze–thaw cycles (−80 °C for 1 h and 60 °C water bath for 2 h per cycle). After centrifugation (10,000× *g* rpm, 10 min) to remove cellular debris, the supernatant was treated with three volumes of ethanol and incubated at 4 °C for 10–12 h. Precipitates were collected by centrifugation (10,000× *g* rpm, 10 min), dissolved in distilled water, and re-centrifuged to eliminate insoluble residues. The extracellular and intracellular polysaccharide content was quantified using the phenol-sulfuric acid method. For melanin analysis, 10 mL of culture was mixed with an equal volume of 1 mol/L NaOH and boiled for 3 h. After cooling to room temperature, the mixture was centrifuged (10,000× *g* rpm, 5 min) to remove biomass. The supernatant pH was adjusted to 2.0 with 1 mol/L HCl, followed by incubation at 70 °C for 14 h. The precipitated melanin was collected by centrifugation and dried to a constant weight at 55 °C [28].

### 2.5. Microbial Community Structure Analysis

The microbial community structure of *T. fuciformis* liquid spawn was analyzed as described by Zhou et al. [29]. Mycelia were collected by centrifugation, and genomic DNA was extracted using the CTAB method. DNA integrity was assessed via 1.0% agarose gel electrophoresis, while concentration and purity were quantified using a NanoDrop 2000 ultra-micro spectrophotometer (Thermo Fisher, Waltham, MA, USA). Microbial conserved regions were amplified by PCR. The bacterial 16S rRNA V3–V4 regions were amplified using the primers 5′-ACTCCTACGGGAGGCAGCA-3′ and 5′-GGACTACHVGGGTWTCTAAT-3′. The fungal ITS1 regions were amplified using the 5′-CTTGGTCATTTAGAGGAAGTAA-3′ and 5′-GCTGCGTTCTTCATCGATGC-3′ [30]. PCR products integrity was verified by 1.0% agarose gel electrophoresis, and qualified samples were submitted to Biomarker Technologies Co., Ltd. (Beijing, China) for Illumina NovaSeq 6000 sequencing. Sequencing libraries were prepared using the VAHTSTM Universal DNA Library Prep Kit (Vazyme, Najing, China), quality-checked with a Qsep-400 analyzer (Bioptic Inc, Changzhou, China), and quantified with a Qubit 3.0 fluorometer (Thermo Fisher, Waltham, MA, USA). Raw sequencing reads were assembled and subjected to quality control to generate Clean Tags. Chimeric sequences were filtered to obtain Effective Tags. OTUs (operational taxonomic units) were clustered at a similarity threshold of ≥97% based on 16S rRNA and ITS1 gene sequences. Shared and unique OTUs were visualized using Venn diagrams in R (version 3.3.1). Alpha diversity indices (Shannon, Simpson, Good’s coverage) were calculated using QIIME 1.9.1 with rarefaction analysis. Differences in community composition and diversity were analyzed in R (v3.6.3), and Venn diagrams were generated using the VennDiagram package (v1.7.3). Taxonomic composition was visualized as percentage stacked bar plots in Microsoft Excel.

### 2.6. Preparation of T. fuciformis Liquid Spawn

To prepare *T. fuciformis* liquid spawn, the *A. stygium* metabolic extract was prepared from post-harvest *T. fuciformis* cultivation substrates. Briefly, 400 g of the substrate was homogenized with 1 L of ultrapure water using a commercial blender (Zhongchong (Jining, China), TJ3). The mixture was subjected to thermal extraction in a boiling water bath for 30 min, followed by filtration to remove solid residues. The filtrate was then adjusted to a final volume of 1 L with ultrapure water, sterilized at 115 °C for 30 min, and stored at 4 °C for subsequent use. This aqueous extract, containing water-soluble metabolites derived from *A. stygium* metabolic activity during *T. fuciformis* cultivation, was designated as the *A. stygium* metabolic extract. Cryopreserved *T. fuciformis* Tr21 spores (stored at −80 °C) were streaked onto PDA plates and incubated at 24 °C until hyphal growth emerged from colony margins. Hyphal tips were then transferred to fresh PDA plates supplemented with 30% (*v*/*v*) *A. stygium* metabolic extract and cultured at 24 °C until mycelia covered approximately one-third of the plate. The resulting culture was used as pure *T. fuciformis* solid spawn. Concurrently, cryopreserved *A. stygium* was revived on PDA plates at 24 °C. Agar plugs (diameter φ = 0.5 cm) from colonies with culture medium darken were transferred to fresh PDA plates to obtain *A. stygium* solid pawn. Subsequently, pure *T. fuciformis* solid spawn plugs (φ = 0.5 cm) were inoculated into 100 mL PDB (potato 200 g/L, glucose 20 g/L, peptone 5 g/L, KH_2_PO_4_ 2 g/L, MgSO_4_ 1 g/L, Thiamine HCl 0.01 g/L) supplemented with 30% *A. stygium* metabolic extract and incubated at 24 °C, 140 rpm to produce pure *T. fuciformis* liquid spawn. Similarly, *A. stygium* solid spawn was inoculated into PDB to prepare *A. stygium* liquid spawn. Physiological parameters were monitored to determine the optimal cultivation period. Finally, liquid spawns of pure *T. fuciformis* and *A. stygium* were mixed at defined ratios to produce the final *T. fuciformis* liquid spawn.

### 2.7. Cultivation Tests

#### 2.7.1. Cultivation Test of Liquefied Spawn

For the *T. fuciformis* cultivation tests, the cultivation medium was composed of cottonseed hull, wheat bran, and gypsum at a ratio of 85:14:1. The moisture content of the cultivation substrate was adjusted to 58–60% (*w*/*w*) using a moisture meter (LXT, Recht (Collingwood, Melbourne, VI, Australia)). A total of 1500 g of the moistened substrate was packed into polyethylene bags (55 × 13 cm). After perforation and sealing with adhesive tape, the bags were autoclaved at 121 °C for 150 min. Once cooled to room temperature, 5 mL of *T. fuciformis* liquefied spawn was aseptically inoculated into each bag through the perforation site. The inoculation sites were then resealed, and the bags were incubated in a fruiting room under standard fruiting management conditions. To evaluate cultivation stability, four consecutive batches were conducted, each consisting of 60 bags. Fruiting performance was assessed based on fruiting yield and ratio. Parallel comparisons with solid spawn controls were carried out to determine yield differences. The fruiting body yield and fruiting ratio were calculated according to the following formula:

$$Yield\;(g/kg)=\frac{Fruiting\;body\;mass(g)}{Dry\;substrate\;mass(kg)}$$

Fruiting body mass: The weight of *T. fuciformis* fruiting body per bag.

Dry substrate (ds) mass: Wet substrate weight per bag × (1 − substrate moisture).

$$Fruiting\;ratio\;(\%)=\frac{Fruiting\;body\;numbers}{Perforation\;numbers}\times 100\%$$

Fruiting body numbers: The total number of fruiting bodies harvested from 60 bags.

Perforation numbers: 60 bags × 3 perforation numbers per bag.

#### 2.7.2. Cultivation Test of Liquid Spawn

Liquid spawns of pure *T. fuciformis* (cultivated for 7 days) and *A. stygium* (cultivated for 3 days) were directly mixed under sterile condition at various volume ratios (5:1, 4:1, 3:1, 2:1, and 1:1, *v*/*v*). For each mixing ratio, 5 mL of the liquid spawn mixture was inoculated into substrate bags following the procedure outlined in Section 2.7.1. Fruiting performance was analyzed to identify the optimal mixing ratio, with solid spawn used as the control. Additionally, industrial-scale tests were also performed to evaluate the practical application of liquid spawn in commercial production.

### 2.8. Statistical Analysis

Statistical analyses were conducted using GraphPad Prism 9 and Origin 2018. Fruiting performance was evaluated based on yield, fruiting ratio, and agronomic traits of fruiting bodies (diameter, convexity, central diameter, and thickness). One-way analysis of variance (ANOVA) was performed using SPSS 22.0. Duncan’s Multiple Range Test was used to determine significant differences (*p* < 0.05) and highly significant differences (*p* < 0.01). Results are presented as mean ± standard deviation.

## 3. Results and Discussion

### 3.1. Preparation of T. fuciformis Liquefied Spawn

**Figure 1:**
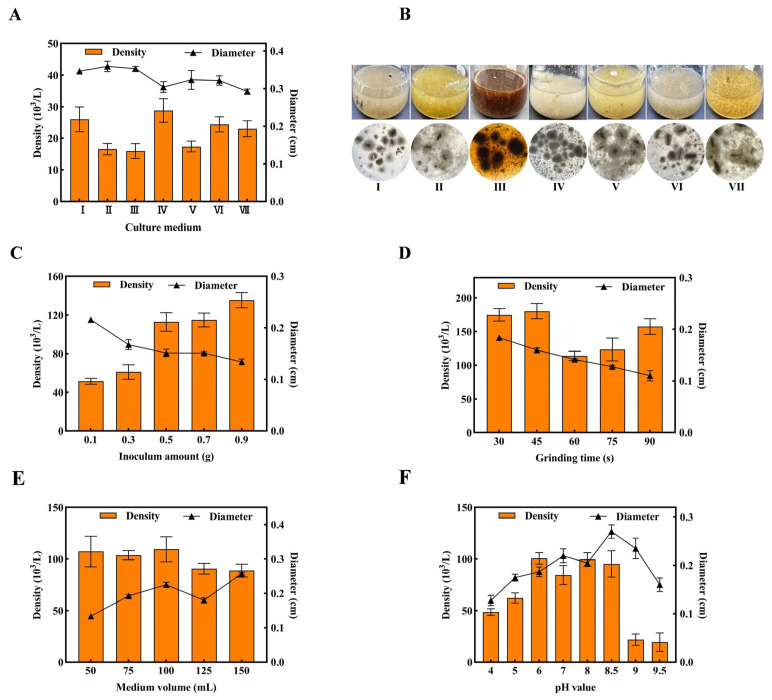
Optimization of *T. fuciformis* liquefied spawn preparation. (**A**) Screening of liquefied media; (**B**) Pellet morphology in different media; (**C**) Inoculum amount; (**D**) Grinding time; (**E**) Medium volume; (**F**) Initial pH value.

In edible mushroom cultivation, inoculum production systems are traditionally categorized into solid inoculum and liquid inoculum methodologies [31]. The liquefied spawn was produced by homogenizing high-quality solid spawn in sterile water, followed by sequential scale-up regeneration. This innovative approach addresses key limitations, including the long spawn running time [14], technical complexity, and high capital costs. The preparation of *T. fuciformis* liquefied spawn was systematically developed. A vigorously growing, contamination-free mother spawn was homogenized to produce a mother suspension. This suspension was then inoculated into seven different liquefied spawn media I-VII for mycelial rehabilitation. As shown in Figure 1A, Medium IV exhibited the highest mycelial pellet density (28.8 × 10^3^/L), followed by Media I (26 × 10^3^/L), VI (24.4 × 10^3^/L), and VII (23 × 10^3^/L). Mycelial pellet diameters remained relatively consistent across all media (0.29–0.35 cm). Media II, V, and VII showed structural friability, characterized by increased free hyphae, while Media III and IV contained abundant insoluble residues due to excessive sawdust and soybean powder (Figure 1B). These results indicated that nutritional conditions strongly influenced mycelial growth [32]. Medium VII was ultimately selected due to its cost-effectiveness and ease of preparation. Key preparation parameters including inoculum amount, grinding time, medium volume, and initial pH value were further optimized. As shown in Figure 1C, increasing inoculum amount from 0.1 g to 0.5 g enhanced mycelial pellet density from 51.4 × 10^3^/L to 112.8 × 10^3^/L. Beyond this point, mycelial pellet counts stabilized with no significant quantitative changes. Mycelial pellet diameter showed the inverse trend, decreasing from 0.22 cm to 0.15 cm. The highest densities were observed at 30 s and 45 s. Prolonged grinding caused hyphal damage, reducing pellet numbers (Figure 1D). Medium volume had a limited impact on liquefied spawn quality. Mycelial pellet densities remained comparable (>100 × 10^3^/L) at volumes between 50 and 100 mL (Figure 1E). Volumes exceeding 100 mL led to oxygen transfer limitations and insufficient hydrodynamic shear stress [33], resulting in reduced pellet density and increased mean diameters. In contrast, medium initial pH exerted a significant regulatory effect on mycelial growth. The maximum pellet density (100.6 × 10^3^/L) was achieved at pH 6.0 within the viable range (pH 6.0–8.5). Extreme acidity or alkalinity (pH < 5.0 or >8.5) inhibited mycelial proliferation. Pellet diameters exhibited pH-dependent expansion (Figure 1F). Considering both yield efficiency and morphological characteristics, the final protocol was established with the following specifications: Medium VII formulation, 0.5 g inoculum amount, 30 s grinding time, 100 mL medium volume, and initial pH 6.0. Under these optimized conditions, high-quality *T. fuciformis* liquefied spawn was successfully produced.

### 3.2. Quality Dynamics of T. fuciformis Liquefied Spawn During the Cultivation Process

**Figure 2:**
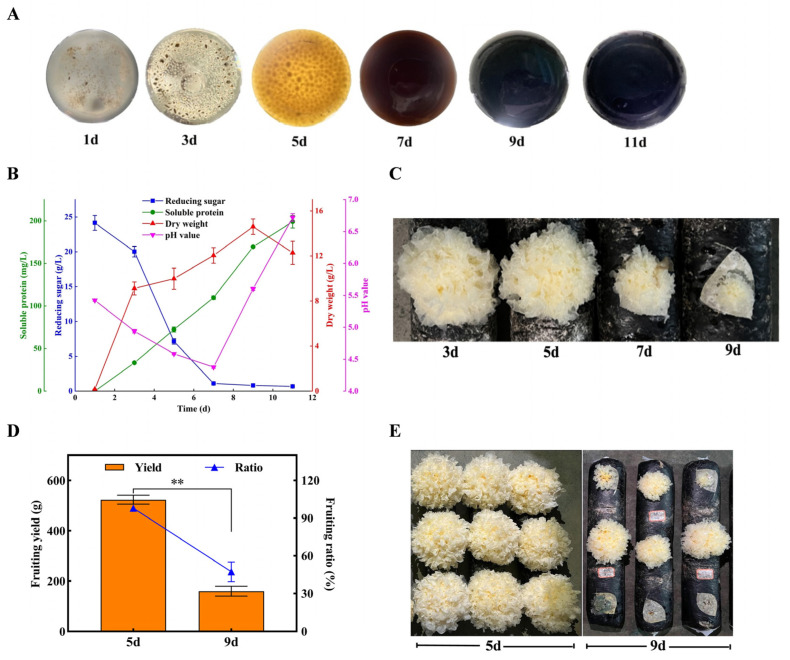
Physiological parameters and cultivation performance of *T. fuciformis* liquefied spawn. (**A**) Morphological evolution; (**B**) Physiological parameter evolution; (**C**) The influence of spawn harvest time on fruiting evolution; (**D**) Yield and fruiting ratio of standard (5d) and browned (9d) *T. fuciformis* liquefied spawn; (**E**) Fruiting body growth performance. ** significance at *p* < 0.01.

Spawn quality plays a critical role in subsequent fruiting yield [34]. Systematic monitoring of physiological changes during liquid cultivation elucidates spawn quality dynamics, establishes cultivation endpoints, and provides a reference for the optimal incubation period [35,36]. Comprehensive analysis of *T. fuciformis* liquefied spawn revealed phase-dependent physiological and morphological patterns. As shown in Figure 2A, the cultivation process exhibited characteristic morphological evolution. Mycelia germinated rapidly, forming ovoid pellets by day 3. It subsequently expanded into spherical pellets as hyphae thickened around the periphery. Chromatic transitions mirrored metabolic activity [37]. The chromatic evolution of the culture medium demonstrated transitioning from initial translucent suspension to pale yellow pigmentation by day 5, progressing through browning phases to complete media blackening at day 9. These visual biomarkers correlated with biochemical transformations, providing critical morphological criteria for assessing spawn maturation stages. Physiological parameter analysis (pH, mycelial dry weight, soluble protein, glucose concentration) in *T. fuciformis* liquefied spawn delineated two metabolically distinct phases (Figure 2B). Post-inoculation glucose catabolism exhibited rapid substrate utilization kinetics, with concentration plummeting from initial 34.2 g/L to 1.10 g/L by day 7, concomitant with biomass accumulation peaking at 14.6 g/L by day 9. Metabolic acidogenesis during glycolysis decreased medium pH to 4.38 by day 7 [38], followed by pH rebound potentially attributable to the utilization of amino nitrogen compounds and the generation of NH_3_/NH_4_^+^ after carbon source depletion [39]. Soluble protein exhibited linear accumulation, reaching 199.08 mg/L at day 11. These results suggest two distinct phases: a nutrient-rich growth phase (0–7 days), characterized by accelerated carbon assimilation and biomass production with concomitant pH decline; and a nutrient-depleted stationary phase (after day 7), marked by carbon starvation, growth stagnation, and pH elevation. The influence of spawn harvest time on fruiting performance was systematically evaluated through cultivation test. As illustrated in Figure 2C, inoculation of pre-browning spawn (3–5 days) supported normal fruiting body morphogenesis. Conversely, post-day-7 spawn under identical cultivation conditions results in significantly smaller fruiting bodies, delayed growth rate, and malformed fruiting bodies. Quantitative comparative analysis of cultivation performance between standard (5d) and browned (9d) *T. fuciformis* liquefied spawn revealed critical performance divergences (Figure 2D,E). The application of standard spawn demonstrated its cultivation performance, yielding 523.4 g/kg ds (dry substrate) with a fruiting ratio of 98.2%, while exhibiting uniform size and plump morphology of fruiting body. Conversely, the browning spawn exhibited severe performance deterioration with yield decline to 159.3 g (69.5% reduction), and fruiting ratio collapse to 47.3%. Meanwhile, fruiting bodies were small, deformed, or unable to initiate primordia. Overall, these findings indicate that the optimal cultivation time for *T. fuciformis* liquefied spawn is 4–5 days. After 7 days, mycelial growth enters a nutrient-depleted stationary phase, during which it begins to metabolize intracellular reserves and extensively secretes secondary metabolites. This physiological shift is accompanied by visible browning of the culture and a significant deterioration in spawn quality. Consequently, *T. fuciformis* yield decreases substantially.

### 3.3. Comparative Analysis of Microbial Community Shifts During Liquefied Spawn Browning

**Figure 3:**
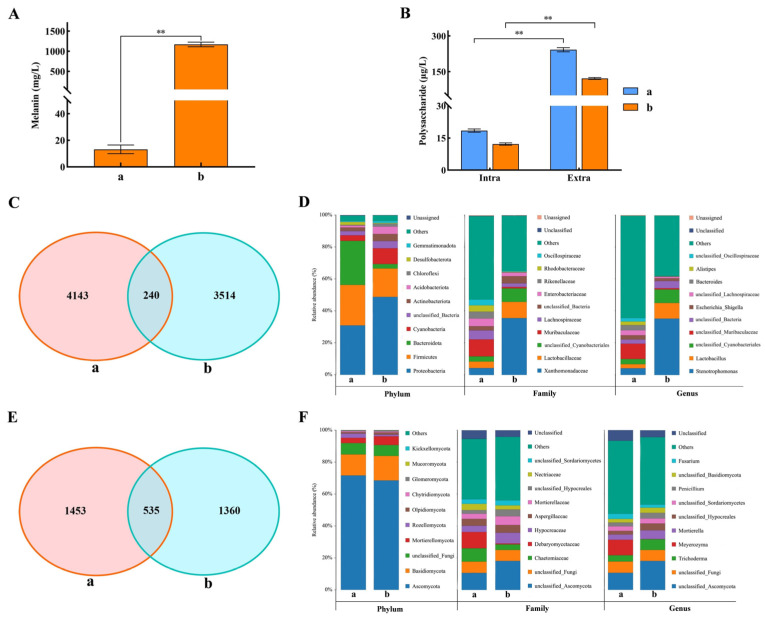
Microbial community dynamics during browning of *T. fuciformis* liquefied spawn. (**A**) Melanin concentration, (**B**) Intra/extra-cellular polysaccharide profiles, (**C**) Venn diagram of bacterial OTUs, (**D**) Bacterial community composition, (**E**) Venn diagram of fungal OTUs, and (**F**) Fungal community composition. Groups a (day-5 standard spawn) and b (day-9 browned spawn) represent pre- and post-browning stages, respectively. ** significance at *p* < 0.01.

*T. fuciformis* spawn inherently comprises two essential fungal components—the *T. fuciformis* strain and its companion *A. stygium* [40]—and latent contamination from solid spawn further complicated the microbial community structure in liquefied spawn [41]. Browning typically occurs around day 7 of cultivation, potentially associated with melanin secretion by *A. stygium*. Polysaccharides represent the primary metabolic products of *T. fuciformis* [42]. Therefore, melanin and polysaccharide contents in liquefied spawn were quantified before and after browning. As illustrated in Figure 3A, melanin content increased dramatically after browning, rising from 13.2 mg/L in the standard liquefied spawn (Group a) to 1168 mg/L in the browned spawn (Group b), representing an 88.5-fold increase. This result indicates that the browning of liquefied spawn is primarily driven by the excessive production of melanin by *A. stygium* [43]. Meanwhile, both intracellular and extracellular polysaccharide contents decreased significantly after browning, by 33.95% and 49.83%, respectively (Figure 3B), suggesting a reduction in the relative abundance of *T. fuciformis* within the microbial community. Microbial community composition was further analyzed. For bacterial communities (Figure 3C,D), a total of 7897 OTUs were identified, among which 240 OTUs (3.04%) were shared between groups. Group a and Group b contained 4143 and 3514 unique OTUs, respectively. At the family and genus levels, *Xanthomonadaceae* (*Stenotrophomonas*) and *Lactobacillaceae* (*Lactobacillus*) exhibited marked increases in relative abundance, with *Stenotrophomonas* rising from 3.7% to 34.3%. *Stenotrophomonas* is a pathogenic species and has been shown to inhibit fungal growth [44]. For fungal communities (Figure 3E,F), a total of 3348 OTUs were identified, with 535 shared OTUs accounting for 15.98% of the total. Group a and Group b contained 1453 and 1360 unique OTUs, respectively. At the phylum level, overall differences were minimal, although *Basidiomycota* and *Mortierellomycota* showed slight increases in Group b. The relative abundance of yeast (*Meyerozyma*) decreased markedly, while populations of potentially harmful fungi, including *Mortierella* and *Trichoderma* were increased. These proliferating pathogens can infect both *T. fuciformis* and *A. stygium* mycelia, directly compromising biological activity and ultimately degrading spawn quality through suppressed metabolic function and structural damage. Notably, *T. fuciformis* and *A. stygium* accounted for 4% and 13% of the fungal community (Group a), respectively, whereas in the browned spawn (Group b), their relative abundances declined to 1% and increased to 16%, respectively. These findings demonstrate that browning is associated with a significant reduction in *T. fuciformis* abundance and a concomitant rise in *A. stygium*, a trend consistent with the observed changes in melanin and polysaccharide levels. In conclusion, browning of *T. fuciformis* liquefied spawn is primarily driven by melanin overproduction from its companion *A. stygium*. Following browning, significant alterations occur in the microbial community structure, characterized by increased abundances of detrimental fungi and pathogenic bacteria. Notably, an imbalance emerges between *T. fuciformis* and *A. stygium*, with *T. fuciformis* abundance declining markedly, while *A. stygium* increases. These microbial and symbiotic shifts constitute a key factor contributing to the substantial decline in spawn quality after browning.

### 3.4. Cultivation Performance of T. fuciformis Liquefied Spawn

**Figure 4:**
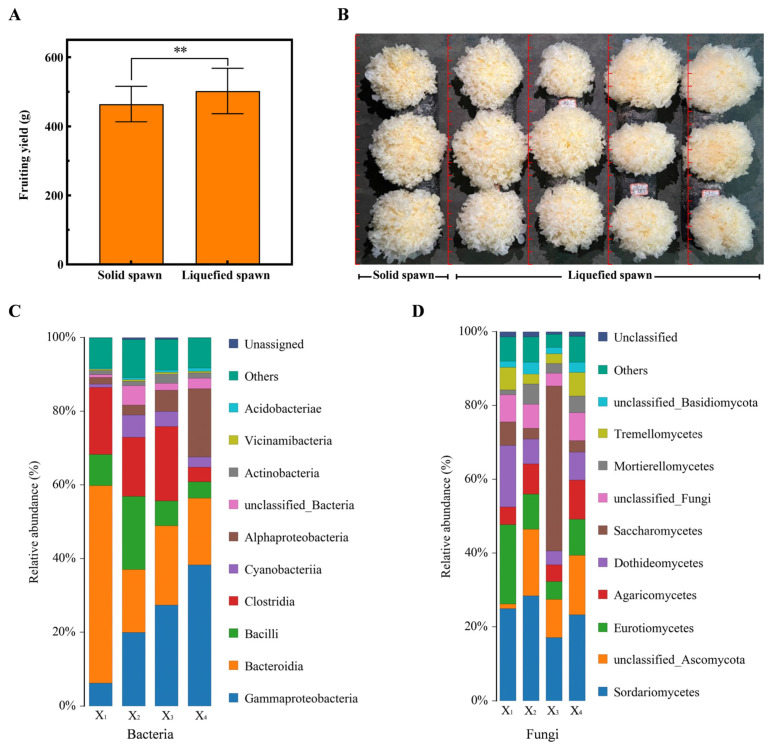
Cultivation performance of *T. fuciformis* liquefied spawn. (**A**) Fruiting body yield, (**B**) Fruiting body growth performance, (**C**) Inter-batch variation in bacterial community structure, (**D**) Inter-batch variation in fungal community structure. ** significance at *p* < 0.01.

*T. fuciformis* liquefied spawn and traditional solid spawn were separately inoculated into cultivation substrates to evaluate and compare their cultivation performance. As shown in Figure 4A,B, the average yield was 464.4 g/kg ds for liquefied spawn and 502.4 g ds for solid spawn, reflecting an improvement of 8.2% (*p* = 0.000806, highly significant difference). Fruiting bodies derived from liquefied spawn exhibited plumper morphology with reduced basal development, thereby enhancing the edible portion. However, variability in fruiting body size remained a limitation. Stability of cultivation performance is a critical parameter for assessing spawn applicability [45]. To evaluate this characteristic, four consecutive cultivation batches were conducted using liquefied spawn. Considerable yield variability was observed across batches, with average yields measuring 549.8 g/kg ds, 489.2 g/kg ds, 510.6 g/kg ds, and 537.9 g/kg ds, respectively. Notably, the yield difference between the first and second batches reached 12.4% (Table 2). Microbial community analysis across the four batches (Figure 4C,D) revealed significant compositional variation. In bacterial communities, the relative abundances of *Gammaproteobacteria*, *Bacteroidia*, and *Bacilli* exhibited pronounced fluctuations. In fungal communities, dominant classes such as *Sordariomycetes*, *Eurotiomycetes*, and *Agaricomycetes* also showed substantial variation. The instability of microbial communities posed a substantial threat to yield consistency in bioprocess systems [29]. Importantly, the first and fourth batches displayed higher abundances of *Tremellomycetes* compared to the second and third batches, which corresponded with the observed differences in yield performance. Consequently, quantitative detection of *Tremellomycetes* abundance should be implemented as a mandatory quality control parameter prior to using *T. fuciformis* liquefied spawn for inoculation. These findings collectively demonstrate that while *T. fuciformis* liquefied spawn provides measurable yield advantages over traditional solid spawn, its complex and unstable microbial community directly contributes to production inconsistency, thereby limiting its commercial-scale application.

### 3.5. Preparation of T. fuciformis Liquid Spawn and Physiological Parameter Assay

**Figure 5:**
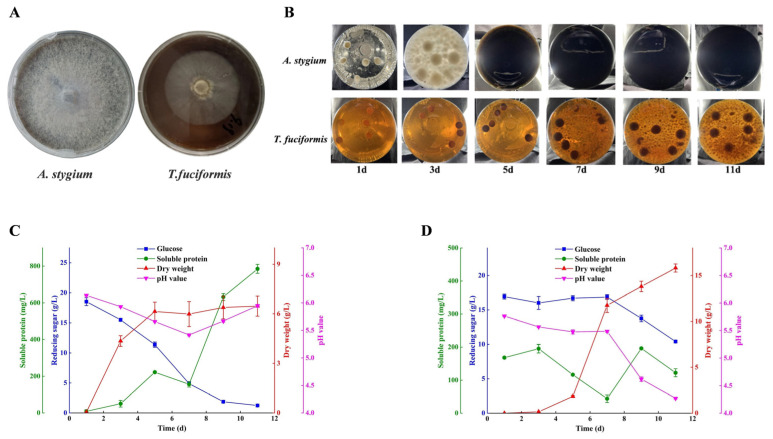
Variations in physiological parameters during the cultivation of pure *A. stygium* and *T. fuciformis* liquid spawn. (**A**) Pure *A. stygium* and *T. fuciformis* mycelia, (**B**) Morphological evolution of liquid spawn, (**C**) Physiological parameter evolution in pure *A. stygium* liquid spawn, (**D**) Physiological parameter evolution in pure *T. fuciformis* liquid spawn.

To address the limitations of *T. fuciformis* liquefied spawn mentioned above, liquid spawn was prepared using pure mycelia of *T. fuciformis* and *A. stygium*. First, solid plate cultures were established. The mycelial growth rates of pure *T. fuciformis* and *A. stygium* on solid plates differed markedly: *A. stygium* grew rapidly, covering the plate within 6–8 days, whereas pure *T. fuciformis* grew slowly, reaching only half the plate diameter after 30 days under identical conditions (Figure 5A). During substrate cultivation, *T. fuciformis* grows slowly, while *A. stygium* rapidly degrades the substrate, supplying abundant nutrients for *T. fuciformis* [46]. When co-inoculated into liquid culture, the substantial difference in mycelial growth rates can lead to an imbalance in their proportions, resulting in malformed or failed fruiting body formation following inoculation onto lignocellulosic substrates. In this study, pure *T. fuciformis* and *A. stygium* mycelia were initially cultivated separately to generate their respective liquid spawns. Physiological parameters were monitored during cultivation to determine the optimal growth periods. As illustrated in Figure 5B, *A. stygium* germinated rapidly after inoculation into PDB. By day 3, extensive mycelial colonization had occurred. This was followed by rapid browning, which occurred earlier than observed in *T. fuciformis* liquefied spawn. Consistent with plate culture observations, *T. fuciformis* mycelia exhibited significantly slower growth, with germination becoming visible around day 5 and dense mycelial growth established by day 9. Physiological parameters of liquid spawns were further analyzed. For *A. stygium* liquid spawn, key physiological indicators including pH, biomass, soluble protein, and glucose concentration are presented in Figure 5C. Glucose was rapidly consumed, decreasing to 1.86 g/L by day 9. Biomass peaked at 6.14 g/L by day 5 and subsequently entered a stable phase. The pH of the medium initially declined before gradually increasing, reaching its lowest value of 5.42 on day 7. Soluble protein content steadily increased to 783.50 mg/L, a trend consistent with that observed in *T. fuciformis* liquefied spawn. In contrast, *T. fuciformis* exhibited slower glucose consumption and mycelial biomass accumulation. During the first 7 days, glucose levels decreased gradually, with a more pronounced decline after day 7, resulting in a final concentration of 10.4 g/L on day 11. Biomass increased slowly to 1.83 g/L within the first 5 days, followed by a rapid increase to 11.74 g/L between days 5 and 7. The pH of the medium followed a pattern similar to glucose consumption, reaching a final value of 4.27 on day 11. Soluble protein content initially declined, reaching a minimum of 43.9 mg/L on day 7 (Figure 5D). In summary, the optimal cultivation period for *A. stygium* liquid spawn is 3 days, whereas that for pure *T. fuciformis* liquid spawn is 7–9 days. The two are subsequently combined at a defined ratio to produce *T. fuciformis* liquid spawn.

### 3.6. Cultivation Test of T. fuciformis Liquid Spawn

**Figure 6:**
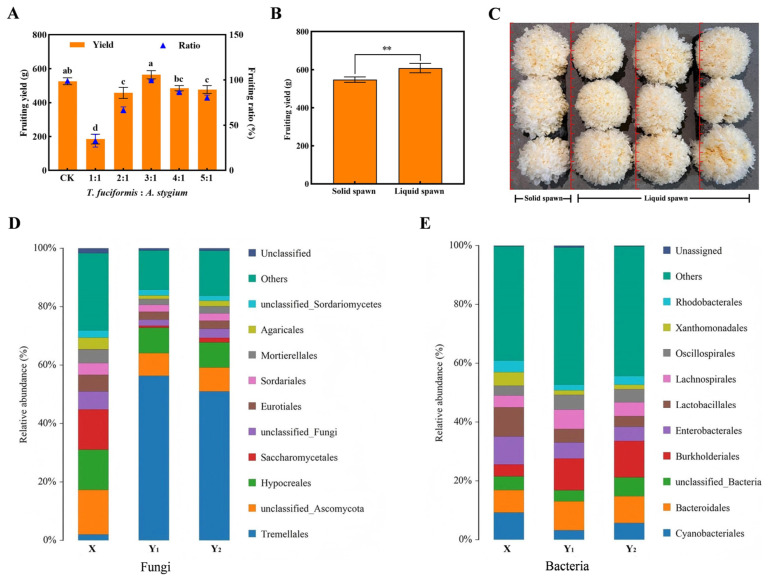
Cultivation performance of *T. fuciformis* liquid spawn. (**A**) Optimization of mixing ratios of pure *T. fuciformis* and *A. stygium* liquid spawns, (**B**) Industrial-scale batch cultivation test, (**C**) Fruiting body growth performance, (**D**) Comparative analysis of fungal community structures, (**E**) Comparative analysis of bacterial community structures. Group X represents *T. fuciformis* liquefied spawn, while Group Y represents *T. fuciformis* liquid spawn. The different lowercase letters denote statistically significant differences (*p* < 0.05). ** Represented significant difference, *p* < 0.01.

Pure *T. fuciformis* and *A. stygium* liquid spawn were prepared and combined to produce *T. fuciformis* liquid spawn. The effect of different mixing ratios on fruiting yield was evaluated. As shown in Figure 6A, increasing the proportions of pure *T. fuciformis* and *A. stygium* liquid spawn initially enhanced both yield and fruiting ratio, which subsequently declined. The highest fruiting body yield (564.4 g/kg ds) and fruiting ratio (98%) were achieved at a mixing ratio of 3:1 (pure *T. fuciformis*: *A. stygium*), representing a 7.3% increase compared to solid spawn (526.0 g). Based on this optimal ratio, large-scale *T. fuciformis* liquid spawn was produced and applied in industrial cultivation using large substrate bags (approximately 1800 g wet substrate per bag). As illustrated in Figure 6B,C, the application of liquid spawn significantly improved both yield and quality. The average yield reached 608.2 g/kg ds, 11.1% higher than that obtained with traditional solid spawn (547.6 g). The resulting fruiting bodies were plump, thick, and uniform, with smaller basal portions, and exhibited superior commercial characteristics. Microbial community analysis revealed that two batches of *T. fuciformis* liquid spawn exhibited similar and stable community structures. In contrast, significant differences were observed between liquefied spawn and liquid spawn, particularly in fungal composition. Liquefied spawn was predominantly composed of *Hypocreales*, *Saccharomycetales*, and *Eurotiales*, whereas liquid spawn was dominated by *Tremellales* and *Hypocreales*, with *Tremellales* representing the absolute dominant group (>50%). Other fungal contaminants were markedly reduced in liquid spawn. Differences in bacterial communities were relatively minor; compared with liquefied spawn, *T. fuciformis* liquid spawn showed decreased abundances of *Xanthomonadales*, *Lactobacillales*, *Enterobacterales*, and *Cyanobacteriales*, but increased abundances of *Burkholderiales* and *Bacteroidales* (Figure 6D,E). Therefore, the stable community composition and high abundance of *Tremellales* contribute to the consistent and reliable cultivation performance of *T. fuciformis* liquid spawn. In summary, the developed *T. fuciformis* liquid spawn demonstrates significant application potential and can fully replace solid spawn for commercial cultivation of *T. fuciformis*.

## 4. Conclusions

This study developed liquid inoculum of *Tremella fuciformis* and evaluated its practical application. The standard liquefied spawn prepared from solid spawn liquification yielded 502.4 g/kg ds, an 8.2% increase over solid spawn, but exhibited unstable cultivation performance due to large fluctuations in microbial community composition and a notably low abundance of *Tremellomycetes* (4%) in the fungal community. In contrast, liquid spawn generated by co-culturing pure mycelia of *T. fuciformis* and *A. stygium* achieved a yield of 608.2 g/kg ds, 11.1% higher than solid spawn, and demonstrated stable and reliable performance. This stability was closely associated with a consistent microbial community structure and the dominance of *Tremellomycetes* (abundance > 50%) in fungal community. Overall, these findings indicate that *T. fuciformis* liquid spawn is more suitable for industrial-scale application than liquefied spawn, as its microbial stability ensures reproducible outcomes and provides a scientific basis for establishing quality control standards in *T. fuciformis* spawn production. Building on these findings, our future research will investigate the dimorphic growth mechanism in *T. fuciformis*, the stimulatory effects of *A. stygium* on *T. fuciformis* mycelial development, and functional roles of key microbial community members. These studies will provide fundamental insights for optimizing high-quality liquid spawn production systems.

## Figures and Tables

**Table 1:** Formulations of liquefied spawn media.

No.	Formulation (g/L)
I	Potato 200, KH_2_PO_4_ 3, Glucose 20, MgSO_4_ 1.5, Thiamine HCl 0.01;
II	Potato 200, Glucose 20, Peptone 5, KH_2_PO_4_ 2, Mg SO_4_ 1, Thiamine HCl 0.01;
III	Potato 200, Sawdust 50, Glucose 20, Peptone 6, KH_2_PO_4_ 0.5, Thiamine HCl 0.01;
IV	Corn flour 10, Soybean flour 7, Glucose 20, Yeast extract 1, Peptone 1, KH_2_PO_4_ 0.5, MgSO_4_ 0.5;
V	Potato 200, Glucose 20, KH_2_PO_4_ 3, Peptone 3, K_2_HPO_4_ 3, MgSO_4_ 1, Thiamine HCl 0.01, Corn flour 1;
VI	Potato 200, Yeast extract 3, Glucose 20, MgSO_4_ 1, KH_2_PO_4_ 1;
VII	Glucose 30, KH_2_PO_4_ 0.5, Peptone 8.3, MgSO_4_·7H_2_O 3, Thiamine HCl 0.01.

**Table 2:** Application stability evaluation of *T. fuciformis* liquefied spawn.

Batch	Yield (g)	Fruiting Ratio (%)	Diameter (mm)	Height (mm)
X_1_	549.8 ± 11.0 a	96	129.0 ± 7.9 a	65.9 ± 2.4 a
X_2_	489.2 ± 11.9 d	98	116.9 ± 14.6 c	60.7 ± 3.1 c
X_3_	510.6 ± 16.2 c	98	118.6 ± 11.8 c	62.1 ± 3.9 bc
X_4_	537.9 ± 5.6 b	98	122.5 ± 9.6 b	64.3 ± 4.3 ab

Note: The different lower-case letters indicate statistically significant difference (*p* < 0.05).

## Data Availability

The original contributions presented in this study are included in the article. Further inquiries can be directed to the corresponding authors.
